# Accumulation of MDSC subsets in renal cell carcinoma correlates with grade and progression free survival, and is associated with intratumoral expression of IL-1β, IL-8 and CXCL5

**DOI:** 10.1186/2051-1426-2-S3-P227

**Published:** 2014-11-06

**Authors:** Yana G Najjar, Patricia A Rayman, Charlie Tannenbaum, Xuefei Jia, Paul Elson, C Marcela Diaz-Montero, Tom Hamilton, Brian Rini, James Finke

**Affiliations:** 1 Department of Hematology-Oncology, University of Pittsburgh Medical Center, Pittsburgh, PA, USA; 2 Department of Solid Tumor Oncology, Cleveland Clinic Foundation, Cleveland, OH, USA; 3 Department of Immunology, Cleveland Clinic, Cleveland, OH, USA

## 

Myeloid derived suppressor cells (MDSC, CD33+CD11b+ HLA-DR ^low/-^) play a major role in tumor-mediated immune evasion and are composed of at least 3 subsets PMN (CD15+), monocytic (CD14+) and lineage-negative (CD15-CD14-), and each has been shown to be significantly increased in some human tumor types and to correlate with metastatic burden, clinical cancer stage and outcome. Less in known about the MDSC subsets that accumulate in tumors such as renal cell carcinoma (RCC) and the cytokines/chemokines involved in their recruitment. Flow cytometry analysis of peripheral blood mononuclear cells (PBMC, n = 20) and nephrectomy samples (n = 39, stage 1-4) showed increased levels of total MDSC in RCC patients compared to normal controls (n = 15), with PMN- and Lin- MDSC subsets dominating in the blood and tumor of RCC patients. Blood levels of total MDSC, PMN-MDSC and Lin-MDSC correlated with tumor grade (p = 0.026, p = 0.006 and p = 0.045, respectively), while blood levels of total MDSC and Lin-MDSC correlated with progression free survival (PFS) in patients with limited stage disease (n = 16, stages 1-3) (HR = 1.35, p = 0.03; HR = 1.45, p = 0.02, respectively). In the tumor, higher PMN-MDSC levels were significantly associated with decreased PFS (n = 29, HR = 1.09, p = 0.011). To assess the role of select chemokines (IL-8, CXCL5, Mip-1α, MCP-1 and Rantes) and of the pro-inflammatory cytokine IL-1β in promoting the accumulation of MDSC within the tumor, these proteins were quantitated in tumor lysates by ELISA and correlated to MDSC frequencies (Spearman correlations). We found a direct correlation between the frequency of PMN-MDSC in the parenchyma and the levels of IL-8 (p < 0.001), CXCL-5 (p < 0.001), and IL-1β (p = 0.029). Frequency of parenchymal Lin- MDSC directly correlated with levels of IL-8 (p = 0.033) and CXCL-5 (p = 0.008), but not IL-1β. In circulation, frequency of total MDSCs directly correlated with IL-1β plasma levels (p = 0.003).

To further define the role of IL-1β in MDSC accumulation within tumors, we overexpressed IL-1β in RENCA and CT26 tumors and compared them to untransfected tumors. Overexpression of IL-1β resulted in enhanced tumor growth and increased frequency of intratumor PMN-MDSC (10.3X in RENCA and 26X in CT26), with a modest increase in intratumor M-MDSC. A large fraction of tumor infiltrating PMN-MDSC expressed CXCR2 (84% in RENCA and 55% in CT26), which is associated with a significant increase in expression of CXCR2 ligands (KC, CXCL5, and MIP2). These results support the idea that IL-1β-mediated induction of select chemokines promotes the accumulation of MDSC, particularly PMN-MDSC, within tumors, resulting in enhanced immune suppression and angiogenesis.

